# Risk factors for the development of acute respiratory distress syndrome in mechanically ventilated adults in Peru: a multicenter observational study

**DOI:** 10.1186/s13054-019-2646-8

**Published:** 2019-12-06

**Authors:** Ena Gupta, Shakir Hossen, Matthew R. Grigsby, Phabiola Herrera, Rollin Roldan, Enrique Paz, Amador A. Jaymez, Eduardo E. Chirinos, Jose Portugal, Rocio Quispe, Roy G. Brower, William Checkley, INTENSIVOS Cohort Study, Roy G. Brower, Francesca Capanni, Maria A. Caravedo, Jorge Cerna, William Checkley, Eduardo E. Chirinos, Long Davalos, Aldo De Ferrari, Joshua A. Denney, Augusto Dulanto, Phabiola Herrera, Amador A. Jaymez, Nicole Mongilardi, Carmen Paredes, Enrique Paz, Maria Alejandra Pereda, Jose Portugal, Rocio Quispe, Rollin Roldan, Navid Shams

**Affiliations:** 1 Division of Pulmonary and Critical Care, School of Medicine, Johns Hopkins University, 1830 E. Monument St, Room 555, Baltimore, MD 21287 USA; 2 Center for Global Non-Communicable Disease Research and Training, School of Medicine, Johns Hopkins University, Baltimore, USA; 3 Servicio de Cuidados Intensivos, Hospital Nacional Edgardo Rebagliati Martins, Lima, Peru; 4 Servicio de Cuidados Intensivos, Hospital Nacional Guillermo Almenara Irigoyen, Lima, Peru; 5 Servicio de Cuidados Intensivos, Hospital Nacional Arzobispo Loayza, Lima, Peru; 6 Servicio de Cuidados Intensivos, Hospital De Emergencias José Casimiro Ulloa, Lima, Peru

**Keywords:** Acute respiratory distress syndrome, Prevention, Critically ill, Mechanical ventilation

## Abstract

**Background:**

Clinical and epidemiological differences between acute respiratory distress syndrome (ARDS) that presents at the initiation of mechanical ventilation [MV] (ARDS at MV onset) and that which develops during the course of MV (ARDS after MV onset) are not well understood. We conducted an observational study in five Peruvian ICUs to characterize differences between ARDS at MV onset and after MV onset and identify risk factors for the development of ARDS after MV onset.

**Methods:**

We consecutively enrolled critically ill patients with acute respiratory failure requiring at least 24 h of mechanical ventilation and followed them prospectively during the first 28 days and compared baseline characteristics and clinical outcomes by ARDS status.

**Results:**

We enrolled 1657 participants on MV (mean age 60.0 years, 55% males) of whom 334 (20.2%) had ARDS at MV onset and 180 (10.9%) developed ARDS after MV onset. Average tidal volume at the initiation of MV was 8.7 mL/kg of predicted body weight (PBW) for participants with ARDS at MV onset, 8.6 mL/kg PBW for those who developed ARDS after MV onset, and 8.5 mL/kg PBW for those who never developed ARDS (*p* = 0.23). Overall, 90-day mortality was 56% and 55% for ARDS after MV onset and ARDS at MV onset, respectively, as compared to 46% among those who never developed ARDS (*p* < 0.01). Adults with ARDS had a higher body mass index (BMI) than those without ARDS (27.3 vs 26.5 kg/m^2^, *p* < 0.01). Higher peak pressure (adjusted interquartile OR = 1.51, 95% CI 1.21–1.88), higher mean airway pressure (adjusted interquartile OR = 1.41, 95% CI 1.13–1.76), and higher positive end-expiratory pressure (adjusted interquartile OR = 1.29, 95% CI 1.10–1.50) at MV onset were associated with a higher odds of developing ARDS after MV onset.

**Conclusions:**

In this study of mechanically ventilated patients, 31% of study participants had ARDS at some point during their ICU stay. Optimal lung-protective ventilation was not used in a majority of patients. Patients with ARDS after MV onset had a similar 90-day mortality as those with ARDS at MV onset. Higher airway pressures at MV onset, higher PEEP, and higher BMI were associated with the development of ARDS after MV onset.

## Background

Acute respiratory distress syndrome (ARDS) is an inflammatory process leading to diffuse edema and life-threatening hypoxemic respiratory failure, which can result from a variety of insults. It is characterized by increased pulmonary vascular permeability, increased lung weight, and loss of aerated lung [1]. Current estimates of mortality range from 26 to 58% with that of low- and middle-income countries (LMICs) in the higher end of this range [2–6]. Indeed, a large, prospective multinational observational study found that low per capita gross national income was associated with lower survival from ARDS [7].

Over the last five decades, extensive basic research has elucidated molecular mechanisms of pathogenesis in ARDS and numerous clinical trials have led to better clinical management for this condition [8–15]. Despite these advances, treatment of ARDS remains mainly supportive. A more preventive approach towards early identification and institution of therapies early in the disease process might have higher success in reducing mortality. The majority of evidence for the diagnosis and management ARDS, however, has stemmed from research conducted in high-income countries. There is a clear need for identification of burden of ARDS and associated morbidity and mortality in resource-poor settings of LMICs [16].

The broad phenotype and etiological causes of ARDS raise the possibility that heterogeneity in ICU characteristics is an important factor in explaining clinical outcomes across settings. These include differences in resources available in the ICU such as health care providers [17], equipment [18], and health care dollars spent per capita [19]. Since being on mechanical ventilation is associated with a higher mortality in LMICs [20], a better characterization of ARDS among mechanically ventilated patients in low-resource settings can help guide changes in critical care delivery. For example, a single-center study by Li et al. reported that changes in critical care structure and delivery were temporally associated with a decrease in the incidence of hospital-acquired ARDS with no change in incidence of ARDS on presentation over an 8-year period [21]. As such, it is crucial to understand risk factors for nosocomial ARDS as a step towards prevention of this condition.

To address these gaps in knowledge, we conducted a multicenter, prospective, longitudinal study of adult, mechanically ventilated patients in five ICUs in Lima, Peru. We sought to characterize the proportions of mechanically ventilated patients with ARDS at mechanical ventilation onset and those who developed ARDS after mechanical ventilation onset while in the ICU. Consequently, we evaluated for differences in etiology, clinical characteristics, mechanical ventilation management, and associated morbidity and mortality in these two groups of patients with ARDS. We also evaluated risk factors for the development of ARDS among those who did not have ARDS at mechanical ventilation onset. With this information, we aimed to identify a high-risk population, health care practices, or factors that help to identify participants who develop ARDS after mechanical ventilation onset while in the ICU.

## Methods

### Study setting

This study was conducted in five ICUs at four public hospitals of the Social Security System (ESSALUD) and Ministry of Health (MINSA) in Lima, Peru. Participating ICUs were selected based on high case volume and willingness to participate. All participating ICUs were closed ICUs with a 24-h in-hospital attending intensivist coverage and with a variable number of critical care fellows and residents depending on the size of the teaching program. Reported annual ICU mortality was similar between units, ranging from 16.7 to 22.9%. None of the ICUs have multidisciplinary rounds or respiratory therapists. There are no established ventilation protocols in use at the Peruvian ICUs; ventilator settings are based on intensivist preference. Details of the characteristics of the participating ICUs and their organization and structure are described elsewhere [22]. We received ethics approval and permission to conduct this study in each of the participating institutions: Hospital Nacional Edgardo Rebagliati Martins, Hospital Nacional Guillermo Almenara Irigoyen, Hospital Nacional Arzobispo Loayza, and Hospital de Emergencias Casimiro Ulloa. Ethics approvals were obtained from the institutional review boards of A.B. PRISMA and ESSALUD Hospital Nacional Edgardo Rebagliati Martins in Lima, Peru, and the Johns Hopkins School of Medicine in Baltimore, USA. We obtained a waiver of written informed consent from all institutions to conduct this observational study.

### Study design

INTENSIVOS (critical in Spanish) is a prospective, observational cohort study. Subjects were consecutively enrolled between December 2010 and October 2013 based on the following eligibility criteria: age ≥ 18 years, at least 24 h of invasive mechanical ventilation in one of the ICUs participating in the study, and enrollment into the study within 48 h of onset of mechanical ventilation. We also assessed disease severity using the SOFA (Sepsis-related Organ Failure Assessment), the SAPS II (Simplified Acute Physiology Score), and the APACHE II and APACHE III (Acute Physiology And Chronic Health Evaluation) scores [23–25]. We obtained demographic, chronic disease, and acute physiological data for all participants meeting eligibility criteria. Participants were followed daily to monitor vital status, clinical and ventilator management, acute physiology, and use of sedation during their ICU stay for up to 28 days in the ICU, until ICU discharge, or death whichever came first. Participants successfully discharged from the ICU were followed for vital status during their inpatient hospital stay. All participants were contacted at 90 days after enrolment to assess their vital status.

### Outcomes

The primary outcome of this analysis was the development of ARDS after mechanical ventilation onset according to the Berlin definition [17]. Although the study was designed before the Berlin definition was published, we were able to combine individual components of the Berlin definition at the analysis stage. Secondary outcomes for this analysis were 90-day mortality, ventilator-free days at 28 days, ICU-free days at 28 days, and hospital-free days at 60 days.

### Definitions

We divided study participants into three categories by their ARDS status during the course of the study: no ARDS at any time during the study period, ARDS at mechanical ventilation onset. and ARDS after mechanical ventilation onset as that which develops while on mechanical ventilation during their ICU stay. We further classified ARDS after mechanical ventilation onset as early if it developed in < 7 days after initiation of mechanical ventilation and late if it developed in ≥ 7 days after initiation of mechanical ventilation.

### Risk factors

Risk factors were defined a priori based on previously published studies showing associations of risk factors for the development of ARDS and ventilator parameters of interest. These included age, sex, APACHE II, SAPS II and SOFA severity scores, body mass index (BMI) [26], tidal volume [27, 28], inspiratory plateau pressure, peak airway pressure, mean airway pressure, driving pressure, and static respiratory system compliance. Plateau pressure was obtained by performing an end-inspiratory hold maneuver on the ventilator that lasted at least 0.5 s. Driving pressure was measured as the difference between plateau pressure and positive end-expiratory pressure (PEEP). Static compliance was measured as tidal volume divided by driving pressure. Fluid balance in the first 24 h was calculated as the difference between total intake and urine output over 24 h.

### Biostatistical methods

We first calculated proportions of mechanically ventilated participants with ARDS at mechanical ventilation onset and with ARDS after mechanical ventilation onset in our cohort. We then estimated the incidence of ARDS after mechanical ventilation onset as the number of mechanically ventilated participants who developed ARDS during their ICU stay divided by the number of ICU days among those without ARDS at mechanical ventilation onset (standardized to 100 ICU days), and used standard methods to calculate a 95% confidence interval.

To visualize the progression of clinical outcomes over time by ARDS status, we graphed the cumulative incidences of death, of achieving unassisted breathing, and of being alive in the hospital and not achieving unassisted breathing. We compared all potential risk factors including ventilator parameters at the initiation of mechanical ventilation and clinical outcomes by ARDS status. We compared groups with chi-square or Fisher exact tests for categorical values, and *t* tests, Wilcoxon rank sum tests or analysis of variance for continuous variables, as appropriate. We used multivariable logistic regression to compare 90-day mortality by ARDS status and used multivariable linear regression to compare ventilator-, ICU-, and hospital-free days by ARDS status adjusted for age, sex, body mass index, APACHE III, and an indicator variable for ICU.

We then used the cohort of participants who did not have ARDS at mechanical ventilation onset to identify predictors of ARDS after mechanical ventilation onset. We conducted single variable logistic regression to model the odds of developing ARDS after mechanical ventilation onset by age, sex, height, BMI, APACHE II, APACHE III, SOFA, SAPS-2, fraction of inspired oxygen (FiO_2_) at the initiation of mechanical ventilation, tidal volume at the initiation of mechanical ventilation, mean airway pressure at the initiation of mechanical ventilation, peak airway pressure at the initiation of mechanical ventilation, plateau pressure at the initiation of mechanical ventilation, driving pressure at the initiation of mechanical ventilation, static respiratory system compliance at the initiation of mechanical ventilation, PEEP at the initiation of mechanical ventilation, PaO_2_/FiO_2_ at the initiation of mechanical ventilation, the difference between actual and predicted body weight (ABW-PBW), and vasopressor use fluid balance in the first 24 h. Finally, given that airway pressures were associated with the development of ARDS after mechanical ventilation onset, we performed separate multivariable logistic regressions for all airway pressures collected at the initiation of mechanical ventilation including mean airway pressure, peak airway pressure, plateau pressure, driving pressure, PEEP, and static respiratory system compliance adjusted for age, sex, body mass index, APACHE III, and an indicator variable for ICU. We ran these adjusted regression models separately to avoid potential collinearity. We also ran separate models for BMI, the difference between actual and predicted body weight, and fluid balance adjusted for age, sex, APACHE III, and an indicator variable for ICU. Finally, we performed subgroup analyses as above to compare baseline characteristics, ventilatory parameters, and clinical outcomes between those who developed early and late ARDS after mechanical ventilation onset.

Analyses were conducted using Stata 12.1 (StatCorp, College Station, Texas) and R (www.r-project.org).

## Results

### Participant characteristics

We screened 1858 participants, of which 116 (6%) did not meet the criteria for enrollment. Of the 1742 enrolled participants, 83 (4.7%) had missing baseline or daily clinical information and 2 (0.1%) were missing a study termination form. A total of 1657 participants were enrolled and had complete data for analysis (Additional file 1: Fig. S1). Mean ± SD age was 60.0 ± 18.9 years, 907 (54.7%) were males, 72.3% were admitted for medical reasons, and the remaining 27.7% were either admitted for trauma or were post-surgical admissions (Table 1). We also plotted the percentage of ICU days by day of mechanical ventilation for which a chest radiograph or arterial blood gas was obtained after mechanical ventilation onset (Additional file 1: Fig. S2).

**Table 1:** Baseline characteristics comparing mechanically ventilated participants with ARDS at mechanical ventilation onset, those of developed ARDS after mechanical ventilation onset, and those who never developed ARDS

Characteristic	Overall	ARDS at mechanical ventilation onset	ARDS after mechanical ventilation onset	No ARDS	*p* value
Number of participants, *n* (%)	1657 (100.0)	334 (20.2)	180 (10.9)	1143 (69.0)	
Being male, *n* (%)	907 (54.7)	191 (57.2)	92 (51.1)	624 (54.6)	0.41
Age in years, mean (SD)	60.0 (18.9)	58.3 (19.4)	59.1 (20.6)	60.7 (18.4)	0.10
Body mass index in kg/m^2^, mean (SD)	26.7 (5.3)	27.0 (5.4)	27.7 (5.6)	26.5 (5.2)	0.02
Height in centimeters, mean (SD)	161.6 (8.8)	161.3 (8.3)	161.5 (9.5)	161.8 (8.8)	0.68
Difference between actual and predicted body weight in kg, mean (SD)	13.7 (14.0)	14.2 (14.3)	16.1 (15.0)	13.2 (13.8)	0.04
Severity scores at the initiation of mechanical ventilation, mean (SD)					
APACHE III	82.7 (28.1)	85.9 (28.6)	82.5 (28.9)	81.8 (27.7)	0.07
SOFA	9.5 (3.5)	10.1 (3.3)	9.3 (3.6)	9.3 (3.5)	< 0.01
SAPS II	54.4 (15.6)	56.0 (16.5)	55.4 (16.0)	53.7 (15.3)	0.04
Comorbidities, *n* (%)					
HIV	29 (1.8)	14 (4.2)	0 (0.0)	15 (1.3)	< 0.01
Tuberculosis	95 (5.7)	19 (5.7)	10 (5.6)	66 (5.8)	0.99
Leukemia	15 (0.9)	6 (1.8)	2 (1.1)	7 (0.6)	0.12
Malignant tumor	122 (7.4)	19 (5.9)	11 (6.2)	52 (4.9)	0.75
Dialysis	122 (7.4)	16 (4.8)	11 (6.1)	95 (8.3)	0.07
COPD	32 (1.9)	7 (2.2)	4 (2.3)	21 (1.8)	0.93
Cirrhosis	63 (3.8)	17 (5.2)	8 (4.5)	38 (3.3)	0.15
Chronic renal disease	251 (15.2)	43 (13.3)	21 (11.8)	187 (16.2)	0.43
Diabetes	254 (15.4)	39 (12.0)	25 (15.4)	190 (16.5)	0.15
Congestive heart failure	153 (9.3)	18 (5.6)	17 (9.6)	118 (10.3)	0.01
Vasopressor use first 24 h, *n* (%)	1002 (60.5)	1002 (60.5)	104 (57.8)	688 (60.2)	0.49
Fluid balance first 24 h, mL (SD)	4474 (2607)	4160 (2192)	4170 (2345)	4515 (2744)	< 0.01
Admission service, *n* (%)					
Medicine	1196 (72.3%)	240 (73.8%)	125 (70.2%)	831 (72.1%)	0.86
Trauma	177 (10.7%)	25 (7.7%)	19 (10.7%)	133 (11.5%)	0.23
Scheduled surgery	58 (3.5%)	14 (4.3%)	3 (1.7%)	41 (3.6%)	0.81
Unscheduled surgery	178 (10.8%)	33 (10.1%)	28 (15.7%)	117 (10.2%)	0.75
Other	46 (2.8%)	13 (4%)	3 (1.7%)	30 (2.6%)	0.58

### Ascertainment of ARDS in the ICU

Of the 1657 mechanically ventilated participants, 514 (31%) met criteria for ARDS during the first 28 days, of which 334 (20.2%) were found to have ARDS at mechanical ventilation onset and 180 (10.9%) developed ARDS after mechanical ventilation onset. The incidence of ARDS after mechanical ventilation onset was 1.07 events per 100 ICU days (95% CI 0.92 to 1.23 events per 100 ICU days). The average ± SD number of days in the ICU before the onset of ARDS after mechanical ventilation onset was 6.5 ± 6.2 days. The majority of participants with ARDS after mechanical ventilation onset (65%, *n* = 117) developed ARDS in < 7 days of their ICU stay.

We summarize demographics, severity of disease, and past medical history by ARDS status in Table 1. Participants with ARDS at mechanical ventilation onset had a similar APACHE III (*p* = 0.63), SAPS II (*p* = 0.50), and SOFA (*p* = 0.37) at the initiation of mechanical ventilation than those with ARDS after mechanical ventilation onset. APACHE III (*p* = 0.75), SAPS II (*p* = 0.19), and SOFA (*p* = 0.98) at the initiation of mechanical ventilation were similar between those with ARDS after mechanical ventilation onset and no ARDS. On average, however, those with ARDS at any point during the study had a higher APACHE III (*p* = 0.05), SAPS II (*p* = 0.01), and SOFA (*p* = 0.01) scores than those who never developed ARDS. BMI was higher in participants with ARDS when compared to participants who never developed ARDS (*p* < 0.01), but similar between those who had ARDS at mechanical ventilation onset and those who developed ARDS after mechanical ventilation onset (*p* = 0.18). Participants with ARDS after mechanical ventilation onset had a greater ABW-PBW than those who never developed ARDS (*p* = 0.01), whereas ABW-PBW was similar to those who had ARDS at mechanical ventilation onset (*p* = 0.16). Participants with ARDS at mechanical ventilation onset had a lower prevalence of congestive heart failure when compared to those with either in-ICU ARDS (< 0.01) or those who never developed ARDS (*p* < 0.01), whereas we did not find a difference between those who developed ARDS after mechanical ventilation onset and those who never developed ARDS (*p* = 0.51). Participants with ARDS at mechanical ventilation onset had a higher prevalence of having HIV when compared to those with either ARDS after mechanical ventilation onset or those who never developed ARDS (*p* < 0.01).

### Ventilator parameters at the initiation of mechanical ventilation

We summarize ventilator parameters at the initiation of mechanical ventilation by ARDS status in Table 4. Average ± SD tidal volume per kilogram of predicted body weight (kg PBW) for mechanical ventilation was 8.5 ± 2.2 mL/kg for the entire cohort. Among the participants with ARDS at mechanical ventilation onset, 89.6% (*n* = 295) were ventilated with tidal volumes > 6 mL/kg PBW, whereas 92.6% (*n* = 165) of those who developed ARDS after mechanical ventilation onset and 91.1% (*n* = 1020) of those who never developed ARDS were initially ventilated with tidal volumes > 6 mL/kg PBW. Plateau pressure was measured in 71% (*n* = 1177) of individuals in this study. Although plateau pressure was ≤ 30 cm H_2_O in a majority of participants (83.5%) at the initiation of mechanical ventilation, 52.2% had driving pressures > 15 cm H_2_O. Plateau pressure at the initiation of mechanical ventilation was higher in participants with ARDS on presentation when compared to others (*p* < 0.001). There was no difference in driving pressure at the initiation of mechanical ventilation between those with ARDS at mechanical ventilation onset and ARDS after mechanical ventilation onset (17.4 cm H_2_O vs 16.4 cm H_2_O, *p* = 0.17). While plateau and driving pressures at the initiation of mechanical ventilation were higher in participants with ARDS after mechanical ventilation onset than in those who never developed ARDS, this increase was not significant (*p* = 0.45 and *p* = 0.45, respectively). Individuals with ARDS on presentation had the highest average set PEEP at the initiation of mechanical ventilation when compared to those with in-ICU ARDS or those who never developed ARDS (*p* < 0.01). All airway pressures collected at the initiation of mechanical ventilation were higher in participants with ARDS at mechanical ventilation onset, followed by participants ARDS after mechanical ventilation onset and lowest in those who never developed ARDS (Table 2).

**Table 2:** Ventilatory parameters at the initiation of mechanical ventilation among mechanically ventilated participants who had ARDS at mechanical ventilation onset, those who developed ARDS after mechanical ventilation onset, and those who never developed ARDS

Variables, mean (SD)	Overall, *n* = 1657	ARDS at mechanical ventilation onset, *n* = 334	ARDS after mechanical ventilation onset, *n* = 180	No ARDS, *n* = 1143	*p* value
FiO_2_	0.47 (0.18)	0.54 (0.19)	0.44 (0.15)	0.45 (0.18)	< 0.001
PEEP, cm H_2_O	7.7 (3.7)	10.1 (4.5)	7.5 (3.4)	7.0 (3.2)	< 0.001
PaO_2_/FiO_2_, mmHg	260.3 (125.7)	201.6 (101.5)	262.4 (135.0)	278.2 (125.5)	< 0.001
Tidal volume, mL	470.9 (98.9)	479.2 (108.7)	468.4 (93.0)	468.9 (96.7)	0.23
Tidal volume, mL/kg PBW	8.5 (2.2)	8.7 (2.3)	8.6 (2.1)	8.5 (2.2)	0.16
Static compliance, mL/cm H_2_O	34.8 (23.9)	33.9 (20.4)	34.7 (22.1)	35.1 (25.2)	0.79
Driving pressure, cm H_2_O	16.4 (5.4)	17.4 (5.8)	16.4 (5.3)	16.1 (5.2)	< 0.01
Mean airway pressure, cm H_2_O	12.6 (4.2)	15.2 (4.6)	12.6 (4.0)	11.9 (3.8)	< 0.001
Peak inspiratory pressure, cm H_2_O	25.1 (6.7)	29.2 (7.0)	25.4 (6.7)	23.9 (6.1)	< 0.001
Plateau pressure, cm H_2_O	24.0 (6.7)	27.2 (7.5)	23.5 (6.0)	23.1 (6.3)	< 0.001

### Clinical outcomes

**Fig. 1:**
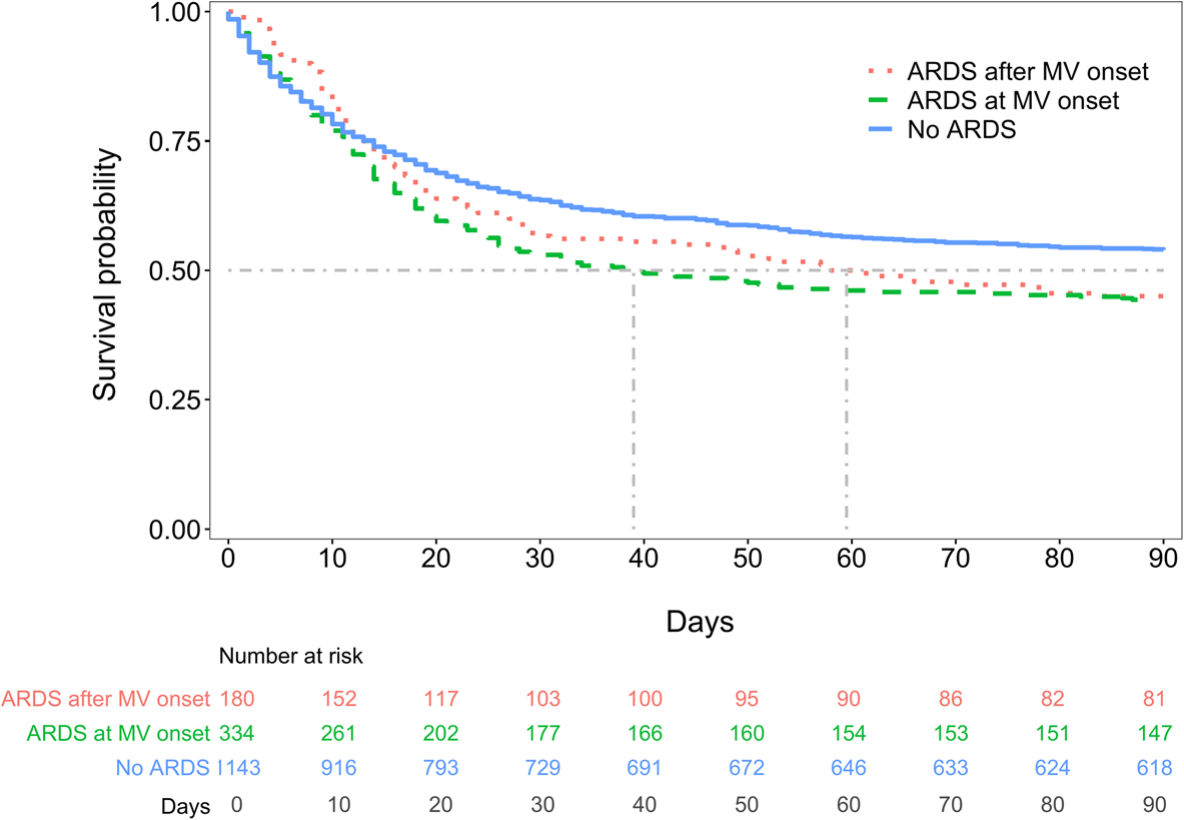
Probability of survival by time in the ICU stratified by ARDS status. Those with no ARDS, shown in blue, had better 90-day survival than those with ARDS at mechanical ventilation [MV] onset (green) or ARDS after mechanical ventilation [MV] onset (red). The participants with ARDS on presentation had a worse survival median survival as compared to those with in-ICU ARDS (40 days vs 60 days) but similar 90-day survival

Mortality at 90 days was 55.6% for study participants who had ARDS anytime during the study as compared to 46.0% in participants who never developed ARDS (*p* < 0.01). Participants with ARDS at mechanical ventilation onset had a similar 90-day mortality than that of participants with ARDS after mechanical ventilation onset in unadjusted (56% vs 55%; *p* = 0.96) and adjusted analysis (*p* = 0.79). Although participants with ARDS at mechanical ventilation onset had a worse median survival than those with ARDS after mechanical ventilation onset (40 days vs 60 days, respectively), both groups had a similar 90-day survival (Fig. 1).

**Fig. 2:**
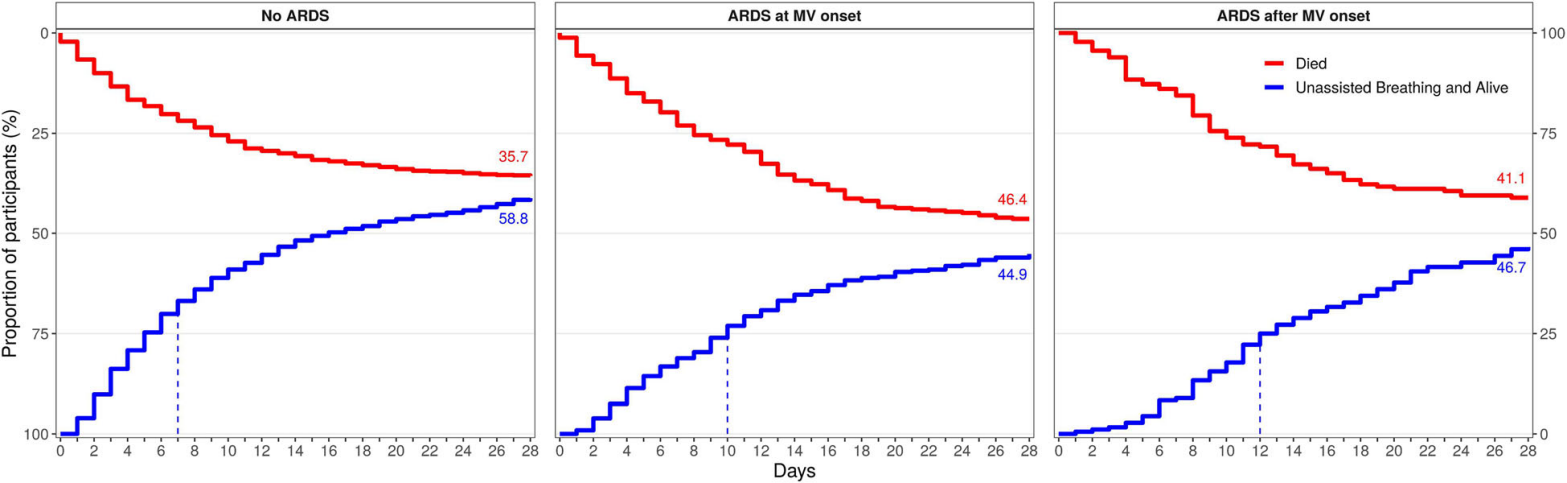
Cumulative incidences of achieving unassisted breathing including being discharged alive and death in the 28-day period of follow-up among the three categories of participants: with no ARDS, ARDS at mechanical ventilation [MV] onset, and who developed ARDS after mechanical ventilation [MV] onset from left to right

We plotted the cumulative percentage of mechanically ventilated participants who achieved unassisted breathing (including those who were discharged alive) and those who died after enrollment in the first 28 days, stratified by ARDS status (Fig. 2). Median time to unassisted breathing was 7 days in no ARDS, 10 days in ARDS at mechanical ventilation onset, and 12 days in ARDS after mechanical ventilation onset. Maximum convergence at 28 days of the cumulative incidences for death and achieving unassisted breathing was seen in those with no ARDS, whereas the least amount of convergence was seen those with ARDS after mechanical ventilation onset. This indicates that participants who developed ARDS after mechanical ventilation onset spent a higher proportion of their time on mechanical ventilation (Fig. 2).

Ventilator-free days, ICU-free days, and hospital-free days were lowest in those with ARDS after mechanical ventilation onset followed by those with ARDS at mechanical ventilation onset and highest in those who never developed ARDS both in single variable (Table 3) and adjusted analyses (Table 4).

**Table 3:** Outcomes among study participants who had ARDS at mechanical ventilation onset, those who developed ARDS after mechanical ventilation onset, and those with no ARDS during their hospital stay

	Overall, *n* = 1657	ARDS at mechanical ventilation onset, *n* = 334	ARDS after mechanical ventilation onset, *n* = 180	No ARDS, *n* = 1143	*p* value
Death 90 days, *n* (%)	810 (48.9%)	187 (56.0%)	99 (55.3%)	524 (46.0%)	< 0.01
Ventilator-free days, mean (SD)	10.1 (10.7)	7.7 (10.0)	6.4 (8.6)	11.3 (10.9)	< 0.001
ICU-free days, mean (SD)	7.3 (8.9)	5.5 (8.1)	4.0 (6.6)	8.4 (9.2)	< 0.001
Hospital-free days, mean (SD)	11.1 (16.6)	9.8 (16.1)	7.3 (13.8)	12.1 (17.1)	< 0.001

**Table 4:** Regression results from analyses looking at differences in outcomes (death at 90 days, ventilator days, ICU-free days, and hospital-free days) by ARDS status

	ARDS at mechanical ventilation onset vs never developed ARDS	ARDS after mechanical ventilation onset vs never developed ARDS	ARDS after mechanical ventilation onset vs ARDS at mechanical ventilation onset
Odds ratio of death at 90 days (95% CI)	1.61 (1.21, 2.14)	1.54 (1.07, 2.20)	0.95 (0.63, 1.45)
Mean difference in ventilator-free days (95% CI)	− 3.82 (− 5.19, − 2.44)	− 5.57 (− 7.32, − 3.82)	− 1.75 (− 3.78, 0.28)
Mean difference in ICU-free days (95% CI)	− 2.95 (− 4.09, − 1.81)	− 4.99 (− 6.43, − 3.54)	− 2.04 (− 3.72, − 036)
Mean difference in hospital-free days (95% CI)	− 3.04 (− 5.21, − 0.87)	− 5.95 (− 8.71, − 3.20)	− 3.05 (− 6.03, − 0.08)

### Factors associated with the development of ARDS after mechanical ventilation onset

**Fig. 3:**
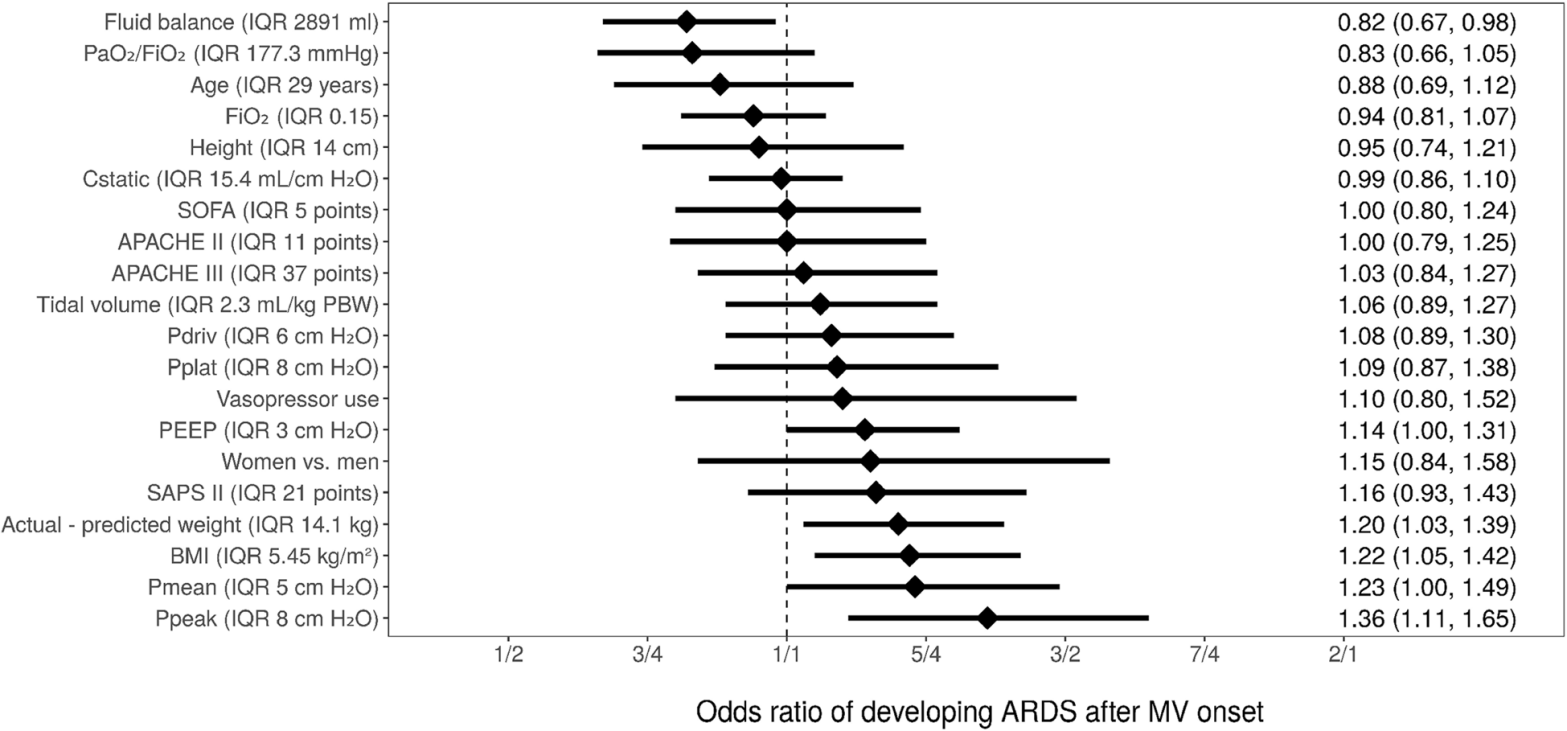
Unadjusted odds ratios of developing ARDS after mechanical ventilation onset vs no ARDS. Interquartile odds ratios are used for continuous variables. Interquartile odds ratios represent an increase in odds of developing ARDS after mechanical ventilation [MV] onset when the variable in question increases from lower 25th quartile to 75th quartile. The diamonds represent the odds ratio and the black bins represent 95% CI intervals. The dotted line is the line of null effect and separates odds in favor of developing in-ICU ARDS on the right and odds against developing in-ICU ARDS to the left of the dotted line. Cstatic, static compliance; *V*_T_, tidal volume per kg of predicted body weight; Pdriv, driving pressure; Pplat, plateau pressure; Pmean, mean airway pressure; Ppeak, peak inspiratory airway pressure

Individual risk factors associated with the risk of developing ARDS after mechanical ventilation onset included a higher BMI, a larger difference between actual and predicted body weight, a higher set PEEP, higher mean airway pressure, higher peak inspiratory airway pressures, and a lower fluid balance in the first 24 h (Fig. 3). In adjusted analyses, fluid balance in the first 24 h was no longer significant (interquartile OR = 0.92, 95% CI 0.74–1.14), whereas BMI (interquartile OR = 1.18, 95% CI 1.01–1.38) and the difference between actual and predicted body weight (interquartile OR = 1.20, 95% CI 1.03–1.39) remained independent predictors for the development of ARDS after mechanical ventilation onset. However, we did not find a difference in tidal volumes at the initiation of mechanical ventilation between participants who developed ARDS following initiation of mechanical and those never developed ARDS. Based on these findings, we tested if tidal volumes at the initiation of mechanical ventilation varied with BMI and predicted body weight. In these analyses, however, we found that tidal volumes at the initiation of mechanical ventilation were not associated with BMI but were positively associated with predicted body weight (Additional file 1: Fig. S3).

**Fig. 4:**
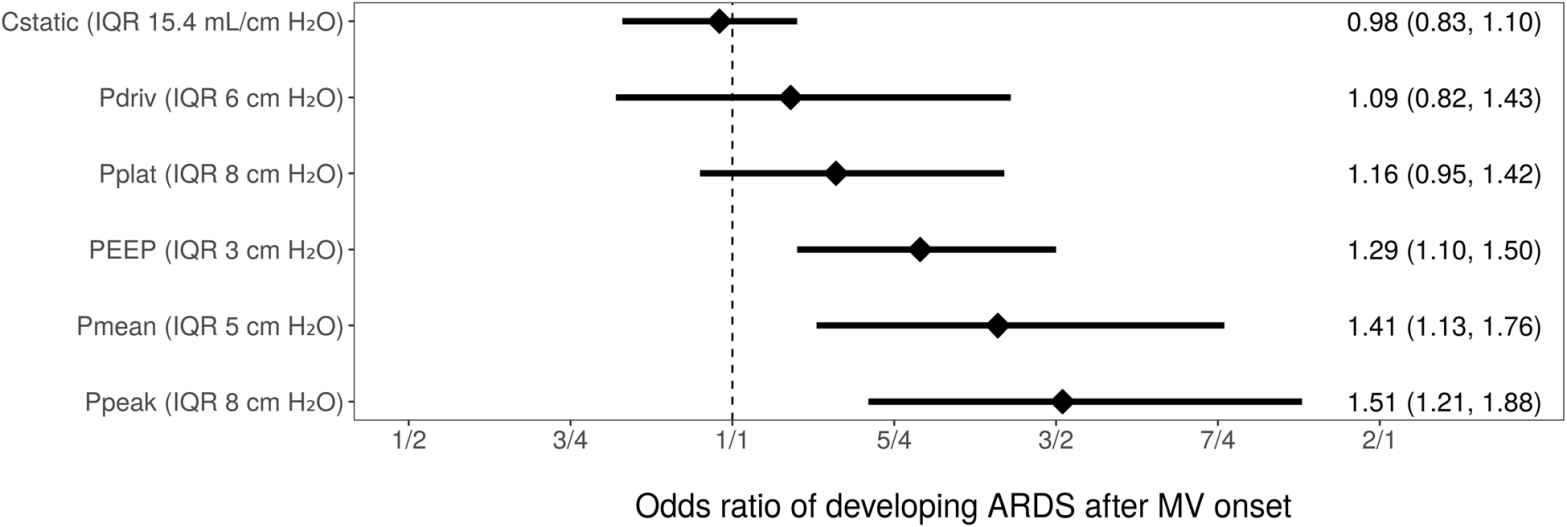
Adjusted odds ratios of developing ARDS after mechanical ventilation onset as compared to no ARDS. All the variables shown were analyzed in separate multivariable models to avoid collinearity. All regression models were adjusted for age, sex, hospital, PEEP, and BMI. Interquartile odds ratios are used for continuous variables. Interquartile odds ratios represent an increase in odds of developing ARDS after mechanical ventilation [MV] onset when the variable in question increases from the lower 25th to the 75th percentile. The diamonds represent the odds ratio, and the black bins represent 95% CI intervals. The dotted line is the line of null effect and separates odds in favor of developing ARDS after mechanical ventilation [MV] onset to the right and odds against developing ARDS after mechanical ventilation [MV] onset to the left of the dotted line. Cstatic, static compliance; Pdriv, driving pressure; Pplat, plateau pressure; Pmean, mean pressure; Ppeak, peak pressure

In adjusted analyses, mean airway pressure, peak airway pressures, and PEEP at the initiation of mechanical ventilation remained significantly associated with the development of ARDS after mechanical ventilation onset (Fig. 4). We also compared risk factors between developed early and late ARDS following initiation of mechanical ventilation, but did not find any differences in age, sex, baseline severity of illness scores, baseline ventilator parameters, and outcomes including 90-day mortality, ventilator-free days, hospital-free days, and ICU-free days in between the two groups in single variable analysis or (Additional file 1: Table S1) adjusted analyses (Additional file 1: Table S2).

## Discussion

In this multicenter study of mechanically ventilated participants in Peru, nearly a third of study participants either had ARDS at mechanical ventilation onset (20.2%) or developed ARDS after the onset of mechanical ventilation (10.9%). A large observational study (LUNG SAFE) conducted in 459 ICUs in 50 countries reported a similar incidence of ARDS during a 4-week period: 23.4% of all patients requiring mechanical ventilation with an incidence density of 1.5 cases of ARDS per 100 ICU days [29]. In another study conducted in a UK university hospital, the incidence of ARDS in patients admitted to the ICU over a 6-month period was 13%, and hospital mortality in patients with ARDS was 42% when compared to 11% in those without ARDS [30]. However, the UK study examined all patients admitted to an ICU whereas LUNG SAFE and our study examined the subset of patients who received mechanical ventilation.

The overall 90-day mortality in our cohort of mechanically ventilated patients was high (49%). Similar mortality rates have been reported in other studies in LMICs [31–36]. 90-day mortality was higher in those with ARDS at mechanical ventilation onset and those with ARDS after mechanical ventilation onset when compared to those who never developed ARDS. We also observed that the majority of study participants were ventilated with > 6 mL/kg PBW. Indeed, the mean tidal volume for patients with ARDS at mechanical ventilation onset was 8.7 mL/kg PBW. This is not in compliance with the ARDS Network trial recommendations of lung-protective ventilation with a tidal volume of ≤ 6 mL/kg predicted body weight and a plateau pressure of 30 cm of H_2_O [37] and highlights an area for improvement. In routine clinical practice, however, non-compliance with ARDS Network ventilation protocols has also been commonly reported in studies conducted in the USA. For example, an observational study conducted in 57 ICUs in the USA found a compliance of 50% with tidal volumes ≤ 6.5 mL/kg PBW in patients with ARDS [38]. In another observational study conducted in Maryland, USA, only 41% of ventilator settings in patients with ARDS were adherent to ARDS network goals [39]. Obstacles to the implementation of lung-protective ventilation need further elaboration in both resource-rich and resource-limited settings to optimize clinical outcomes.

Another area for improvement is lung recruitment. While average peak and plateau pressures were < 30 cm H_2_O, average driving pressures were > 15 cm H_2_O. This can be due to less than optimal lung recruitment, i.e., inadequate application of PEEP. The average set PEEP in this cohort of mechanically ventilated patients was 7.7 cm H_2_O. Although PEEP was higher in those with ARDS on presentation, higher levels of PEEP may actually be needed given high driving pressures. Lower driving pressure, irrespective of tidal volume, is associated with lower mortality in ARDS [40]. Therefore, individualized titration of PEEP through measurement of driving pressure and plateau pressure may improve clinical outcomes [41]. This along with low tidal volume ventilation can facilitate achieving lower driving pressures. While driving pressure at the initiation of mechanical ventilation was not associated with the development of ARDS after mechanical ventilation onset in our study, it is a known predictor of mortality in ARDS [29, 42].

We identified potential factors at the initiation of mechanical ventilation which were associated with the development of ARDS after mechanical ventilation onset. Among the ventilator-related risk factors, higher mean airway pressures, higher peak airway pressures, and higher set PEEP at the initiation of mechanical ventilation were all associated with a higher incidence of ARDS after mechanical ventilation onset in adjusted analyses. The association between higher airway pressures (peak, mean airway, and PEEP) and ARDS development could reflect lung injury, but it could also reflect less-compliant lungs that are sicker and more prone to ARDS development. Indeed, high airway pressures have been linked with early barotrauma and higher mortality [43]. In fact, peak inspiratory airway pressure was also identified to be a potential target to lower mortality in an ancillary analysis from the LUNG SAFE study [43]. High inspiratory flow, for a given plateau pressure or strain, may contribute to ventilator-induced lung injury likely by locally intensified concentration of stress [44, 45]. Higher mean airway pressures may also reflect patients with higher airway resistance, lower compliance, and more elevated respiratory rates which could reflect a greater dead space, all of which can identify a group of sicker patients who are more likely to get ARDS. Mean airway pressure reflects mean alveolar pressure throughout an entire respiratory cycle, which may better reflect the power applied to the lung and risk for injury throughout tidal breathing. Targeting peak or mean airway pressures, therefore, may play a role in preventing ARDS after mechanical ventilation onset.

In contrast, we did not find an association between the development of ARDS after mechanical ventilation onset and either plateau pressures or driving pressures at the initiation of mechanical ventilation, two well-described predictors of ARDS mortality. Nonetheless, both plateau pressure and driving pressure at the initiation of mechanical ventilation were higher in patients with ARDS at the start of mechanical when compared to those who developed ARDS after mechanical ventilation onset or never developed ARDS. There are a couple of possibilities that may explain why we did not find an association between the development of ARDS after mechanical ventilation onset and either plateau of driving pressures at the initiation of mechanical ventilation. First, plateau or driving pressure may take longer to rise in relation to the time of onset of ARDS compared to peak or mean airway pressure. Therefore, plateau or driving pressures measured at the initiation of mechanical ventilation may not capture this relationship. Mean or peak airway pressures may be elevated earlier than plateau or driving pressures in patients who go on to develop ARDS later in the course of mechanical ventilation because physicians may set a higher inspiratory flow rate in response to compensatory tachypnea commonly observed in mechanically ventilated patients with metabolic acidosis resulting from sepsis or septic shock, pancreatitis, or trauma, all of which are well-known risk factors for ARDS. A set higher inspiratory flow rate will result in higher peak airway pressures whereas plateau or driving pressures are minimally affected [46]. Patients with sepsis may also have circulatory inflammatory mediators which causes bronchoconstriction and consequently higher airway resistance [47–49]. This also results in higher peak airway pressures whereas plateau or driving pressures are minimally affected. Patients may also have pulmonary edema that precedes radiographic changes before it causes flooding of alveoli, but which affects conducting airways and increases airway resistance [50]. Second, we had fewer measurements of plateau or driving pressures when compared to peak or mean airway pressure which could have affected our ability to measure a statistically significant difference. We may have had fewer measurements of plateau pressure or driving pressure because they can be cumbersome or difficult to achieve, as it requires a half second inspiratory hold on a ventilator to calculate driving pressure. In our study, nearly 30% (480) of the patients were missing a plateau pressure recording at the initiation of mechanical ventilation. Similarly, in the multinational LUNG SAFE study, plateau pressure was available in only 40% of patients with ARDS, again showing a deficiency of measurement of plateau pressure worldwide in the management of ARDS [29]. It is well established from clinical and animal studies that presumptive application of protective ventilation with low tidal volume to keep plateau pressure < 30 cm H_2_O can reduce the incidence of ARDS [51, 52].

Among patient-related risk factors, higher BMI in participants who did not have ARDS on presentation was significantly associated with the development of ARDS after mechanical ventilation onset. The association between BMI and development of ARDS after mechanical ventilation onset was not to differences in tidal volume. However, it was associated with a statistically significant difference between actual and predicted body weight. This suggests that excess adipose tissue in obese individuals may predispose them to an elevated risk for developing ARDS. Several mechanisms have been proposed for this association in previously published studies like obesity-induced adipokine imbalance [53], obesity-induced endoplasmic reticulum stress-causing endothelium dysfunction [54], chronic inflammatory state of obesity, and elevated neutrophils and cytokines that induce upregulation of adhesion molecules on lung epithelium and enhanced susceptibility to injury [55]. This association has previously been demonstrated in both animal and human studies and is not explained by initial ventilator settings in these individuals.

Our study has some potential shortcomings. First, both higher airway pressure at mechanical ventilation onset and subsequent development of ARDS after mechanical ventilation onset could reflect an underlying severe lung injury rather than a causal effect. Regardless, they serve as an indicator of the development of ARDS in the future, in a cohort of patients who initially do not meet the criterion for ARDS and hence provide an opportunity for early intervention. In addition, the association was adjusted for baseline severity of illness score APACHE III accounting for some of the disease severity at presentation. Second, our study was designed before the publication of the Berlin definition, but the collection of necessary information allowed for the addition of parameters like PEEP to the existing definition to make the criteria more aligned with the Berlin definition. Third, we did not have information about the potential etiology of injury that could have led to the development of ARDS. This precluded us from calculating a Lung Injury Prevention Score (LIPS) at baseline which has been studied to identify those at risk for acute lung injury [56]. Choosing mechanically ventilated participants helped us to enrich our cohort with individuals who are at the highest risk of developing ARDS and enabled us to isolate iatrogenic risk factors in mechanically ventilated individuals. Overall, the observational design allowed us to study current practices and incidence of disease and identify gaps and areas of improvement. Fourth, because this is an observational study, we did not dictate how frequently physicians obtained chest X-rays or arterial blood gases while on mechanical ventilation. This may result in an underestimation of the ARDS incidence after mechanical ventilation onset. Finally, our study was conducted between 2010 and 2013, and changes in the management of sepsis and mechanical ventilation could affect how our findings are applicable today. Replication of our findings in more recent cohorts is necessary.

## Conclusions

ARDS after the onset of mechanical ventilation is common in mechanically ventilated adults, and the associated mortality is equally high as those who are initially admitted with ARDS. Hospital-free days and ICU-free days were lowest among patients with ARDS after mechanical ventilation onset. In this cohort of mechanically ventilated patients in Peru, optimal lung protection ventilation was not used and driving pressures were commonly high. Risk factors for the development of ARDS after mechanical ventilation onset include a high body mass index and the presence of high airway pressures (peak, mean airway, and positive end-expiratory pressures) early in the course of mechanical ventilation. Future studies need to validate the use of these early factors to identify mechanically ventilated patients at risk for developing ARDS after initiation of mechanical ventilation.

## Supplementary information

**Additional file 1: Table S1.** Baseline Characteristics, ventilatory parameters and outcomes in participants with early ARDS (< 7 days) and late ARDS (≥ 7 days) after mechanical ventilation onset. **Table S2.** Adjusted outcomes among study participants comparing early ARDS (< 7 days) and late ARDS (≥ 7 days) after mechanical ventilation onset. All regression models are adjusted for age, sex, hospital and APACHE III. **Figure S1.** Study Flowchart. **Figure S2.** Percentage of ICU-days when a chest radiograph and an arterial blood gas were obtained for all days after mechanical ventilation onset. **Figure S3.** Scatter plot of tidal volume (y-axis) and body mass index (x-axis) on the left and tidal volume (y-axis) and predicted body weight (x-axis) on the right.

## Data Availability

The datasets used and/or analyzed during the current study are available from the corresponding author on request.

## Abbreviations

ARDS: Acute respiratory distress syndrome
ICU: Intensive care unit
LMICs: Low- and middle-income countries
BMI: Body mass index
PEEP: Positive end-expiratory pressure
AUC: Area under the receiver operating characteristic curves
APACHE: Acute Physiology and Chronic Health Evaluation
SOFA: Sequential Organ Failure Assessment
SAPS: Simplified Acute Physiology Score
HIV: Human immunodeficiency virus
PBW: Predicted body weight
LIPS: Lung Injury Prevention Score
