# Supplementary material for: Risk factors for the development of acute respiratory distress syndrome in mechanically ventilated adults in Peru: a multicenter observational study
Source: Crit Care. 2019 Dec 6;23:398. doi: 10.1186/s13054-019-2646-8 (PMC6898929; doi:10.1186/s13054-019-2646-8)
Supplement: Supplementary file 1 — Additional file 1: Table S1. Baseline Characteristics, ventilatory parameters and outcomes in participants with early ARDS (< 7 days) and late ARDS (≥ 7 days) after mechanical ventilation onset. Table S2. Adjusted outcomes among study participants comparing early ARDS (< 7 days) and late ARDS (≥ 7 days) after mechanical ventilation onset. All regression models are adjusted for age, sex, hospital and APACHE III. Figure S1. Study Flowchart. Figure S2. Percentage of ICU-days when a chest radiograph and an arterial blood gas were obtained for all days after mechanical ventilation onset. Figure S3. Scatter plot of tidal volume (y-axis) and body mass index (x-axis) on the left and tidal volume (y-axis) and predicted body weight (x-axis) on the right. [file 13054_2019_2646_MOESM1_ESM.pdf]

Online supplement for "Risk factors for the development of acute respiratory distress syndrome in mechanically ventilated adults in Peru: A multicenter observational study"

Ena Gupta MD, Shakir Hossen MBBS MSPH, Matthew R Grigsby MSPH, Phabiola Herrera MD, Rollin Roldan MD, Enrique Paz MD, Amador A Jaymez MD, Eduardo E Chirinos MD, Jose Portugal MD, Rocio Quispe MD, Roy G Brower MD, William Checkley MD PhD; INTENSIVOS Cohort Study

Table S1: Baseline characteristics, ventilatory parameters and outcomes in participants with early ARDS (<7 days) and late ARDS (≥ 7 days) after mechanical ventilation onset.

	**Early ARDS after mechanical ventilation onset**	**Late ARDS after mechanical ventilation onset**	**Overall**
Number of participants, n (%)	117 (65%)	63 (35%)	180 (100%)
Being male, n (%)	60 (51.3%)	32 (50.8%)	92 (51.1%)
Death at 90 days, n (%)	64 (55.2%)	35 (55.6%)	99 (55.0%)
Age in years, mean (SD)	58.4 (19.2)	60.4 (23.0)	59.1 (20.6)
Body mass index in kg/m^2, mean (SD)	27.7 (5.4)	27.7 (5.9)	27.7 (5.6)
APACHE II, mean (SD)	24.2 (8.3)	23.2 (7.3)	23.8 (7.9)
APACHE III, mean (SD)	84.6 (29.4)	78.6 (27.7)	82.5 (28.9)
SOFA, mean (SD)	9.6 (3.7)	8.8 (3.5)	9.3 (3.6)
SAPS II, mean (SD)	56.5 (16.9)	53.3 (14.2)	55.4 (16.0)

Fluid balance in mL, mean (SD)	4334.2 (2509.8)	3873.4 (1983.7)	4172.9 (2343.8)
Ventilator-free days, mean (SD)	7.4 (9.2)	4.6 (7.1)	6.4 (8.6)
ICU free days, mean (SD)	5.0 (7.3)	2.1 (4.7)	4.0 (6.6)
Hospital free days, mean (SD)	8.4 (14.9)	5.2 (11.2)	7.3 (13.8)
FiO_2 in %, mean (SD)	44.7 (14.8)	42.4 (15.6)	43.9 (15.0)
PEEP, mean cm H_2O (SD)	7.5 (3.5)	7.6 (3.4)	7.5 (3.4)
PaO_2/FiO_2 in mmHg, mean (SD)	247.9 (113.9)	289.1 (165.1)	262.4 (135.0)
Tidal volume in mL, mean (SD)	478.6 (100.5)	449.6 (74.2)	468.4 (93.0)
Static compliance in mL/cm H_2O, mean (SD)	35.0 (25.5)	34.1 (12.4)	34.7 (22.1)
Driving pressure in cm H_2O, mean (SD)	16.3 (5.3)	16.7 (5.5)	16.4 (5.3)
Mean airway pressure in cm H_2O, mean (SD)	12.6 (4.0)	12.6 (3.9)	12.6 (4.0)
Peak inspiratory pressure cm H_2O, mean (SD)	25.4 (6.7)	25.5 (6.8)	25.4 (6.7)
Plateau pressure in cm H_2O, mean (SD)	23.7 (6.1)	23.2 (5.7)	23.5 (6.0)
Tidal volume/predicted body weight in mL per kg/m^2, mean (SD)	8.7 (2.1)	8.3 (2.0)	8.6 (2.1)

Table S2: Adjusted outcomes among study participants comparing early ARDS (<7 days) and late ARDS (≥ 7 days) after mechanical ventilation onset. All regression models are adjusted for age, sex, hospital and APACHE III.

	**Late ARDS (≥ 7 days) vs early ARDS (<7 days) after mechanical ventilation onset**
Odds ratio of death at 90 days	1.19 (0.61, 2.36)
Mean difference in ventilator-free days	-3.09 (-5.76, -0.43)
Mean difference in ICU free days	-3.02 (-4.97, -1.06)
Mean difference in Hospital free days	-3.88 (-8.01, 0.26)

Table S3: Ventilatory parameters at the initiation of mechanical ventilation among mechanically ventilated participants who had ARDS at mechanical ventilation onset or developed ARDS after mechanical ventilation onset.

Variables, mean (SD)	ARDS at mechanical ventilation onset n=334	ARDS after mechanical ventilation onset n=180	p-value
FiO_2	0.54 (0.19)	0.44 (0.15)	<0.001
PEEP, cm H_2O	10.1 (4.5)	7.5 (3.4)	<0.001
PaO_2/FiO_2, mmHg	201.6 (101.5)	262.4 (135.0)	<0.001
Tidal volume, mL	479.2 (108.7)	468.4 (93.0)	0.26
Tidal volume, mL/kg PBW	8.7 (2.3)	8.6 (2.1)	0.513
Static compliance, mL/cm H_2O	33.9 (20.4)	34.7 (22.1)	0.723
Driving pressure, cm H_2O	17.4 (5.8)	16.4 (5.3)	<0.101
Mean airway pressure, cm H_2O	15.2 (4.6)	12.6 (4.0)	<0.001
Peak inspiratory pressure, cm H_2O	29.2 (7.0)	25.4 (6.7)	<0.001
Plateau Pressure, cm H_2O	27.2 (7.5)	23.5 (6.0)	<0.001

Figure S1: Study Flowchart

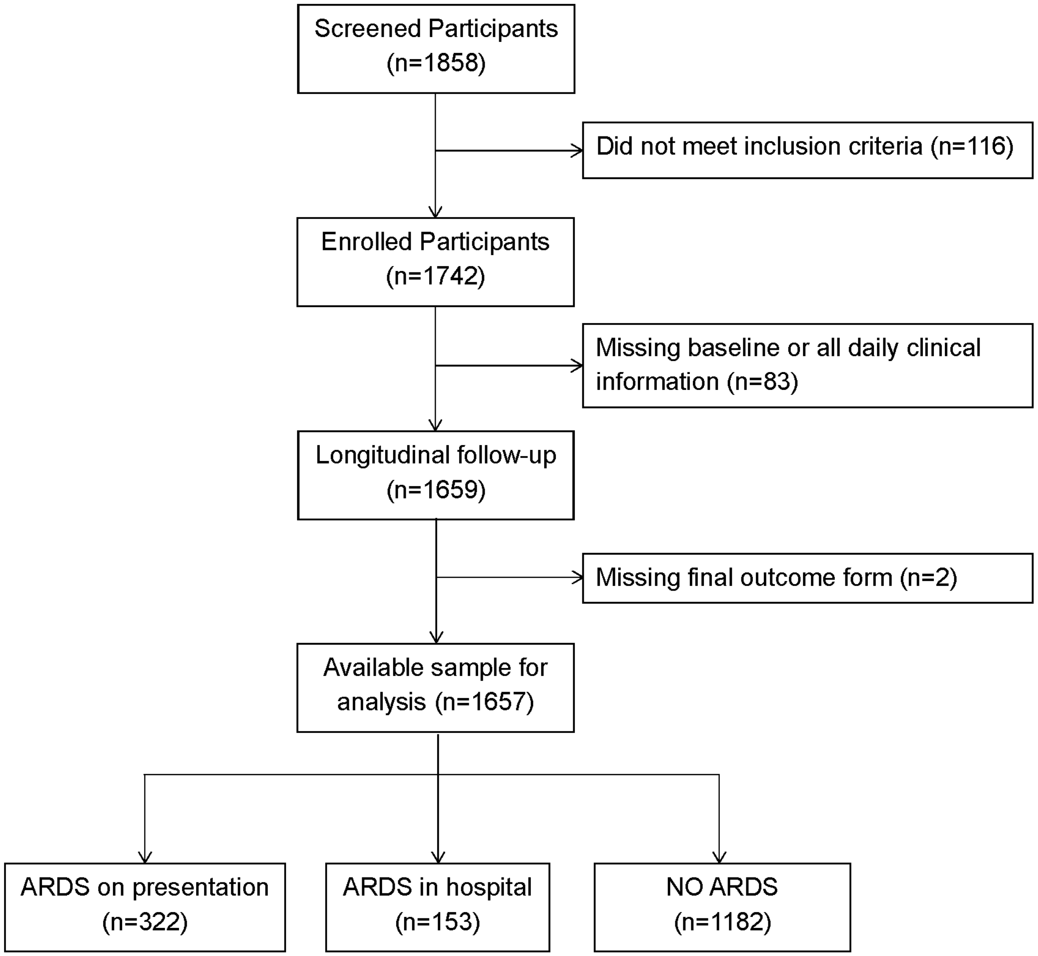

Figure S1

Figure S2: Percentage of ICU-days when a chest radiograph and an arterial blood gas were obtained for all days after mechanical ventilation onset.

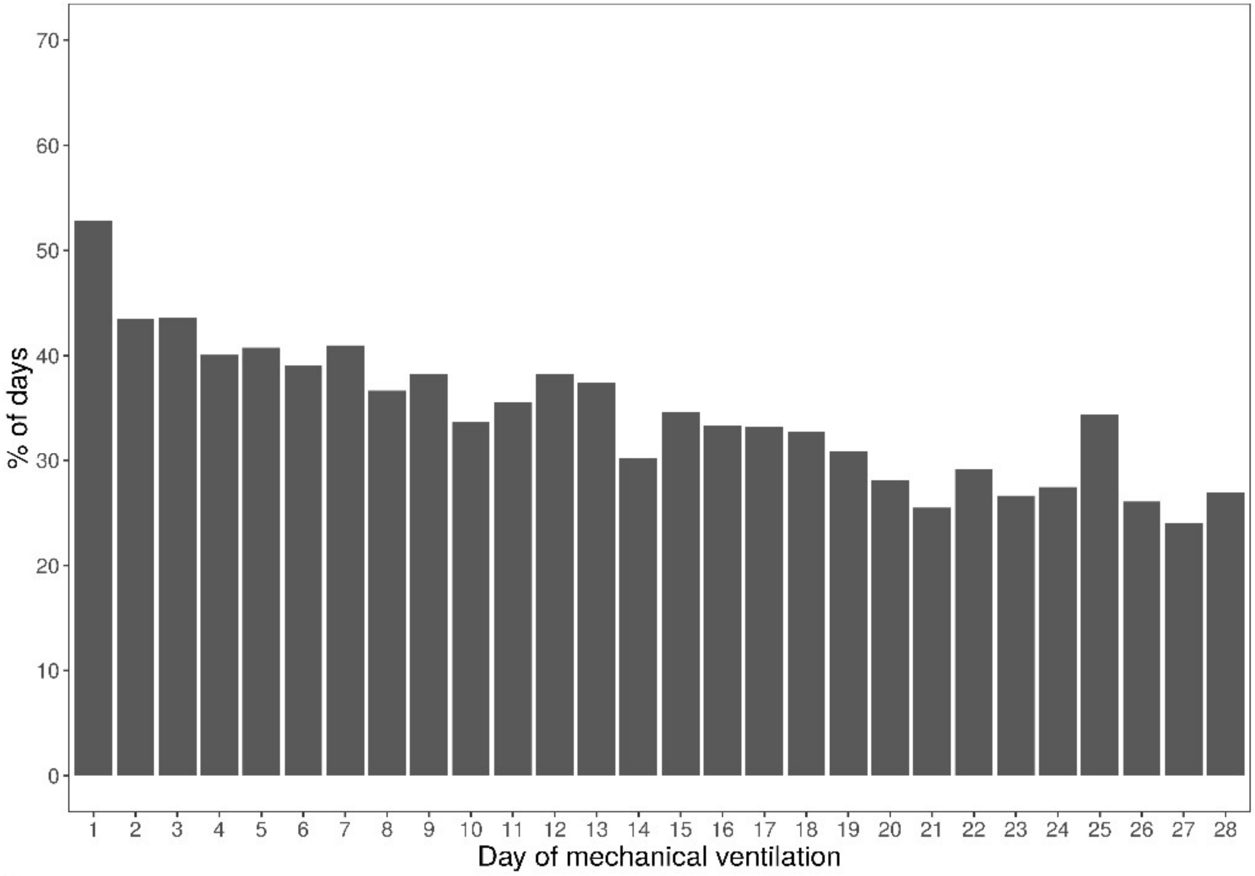

Figure S2

Figure S3: Scatter plot of tidal volume (y-axis) and body mass index (x-axis) on the left and tidal volume (y-axis) and predicted body weight (x-axis) on the right. The solid blue line represents fitted values of tidal volume across body mass index and predicted body weight, respectively. No variation was seen in tidal volume across body mass index (p=0.1) whereas a linear relationship was seen between tidal volume and predicted body weight (p<0.001).

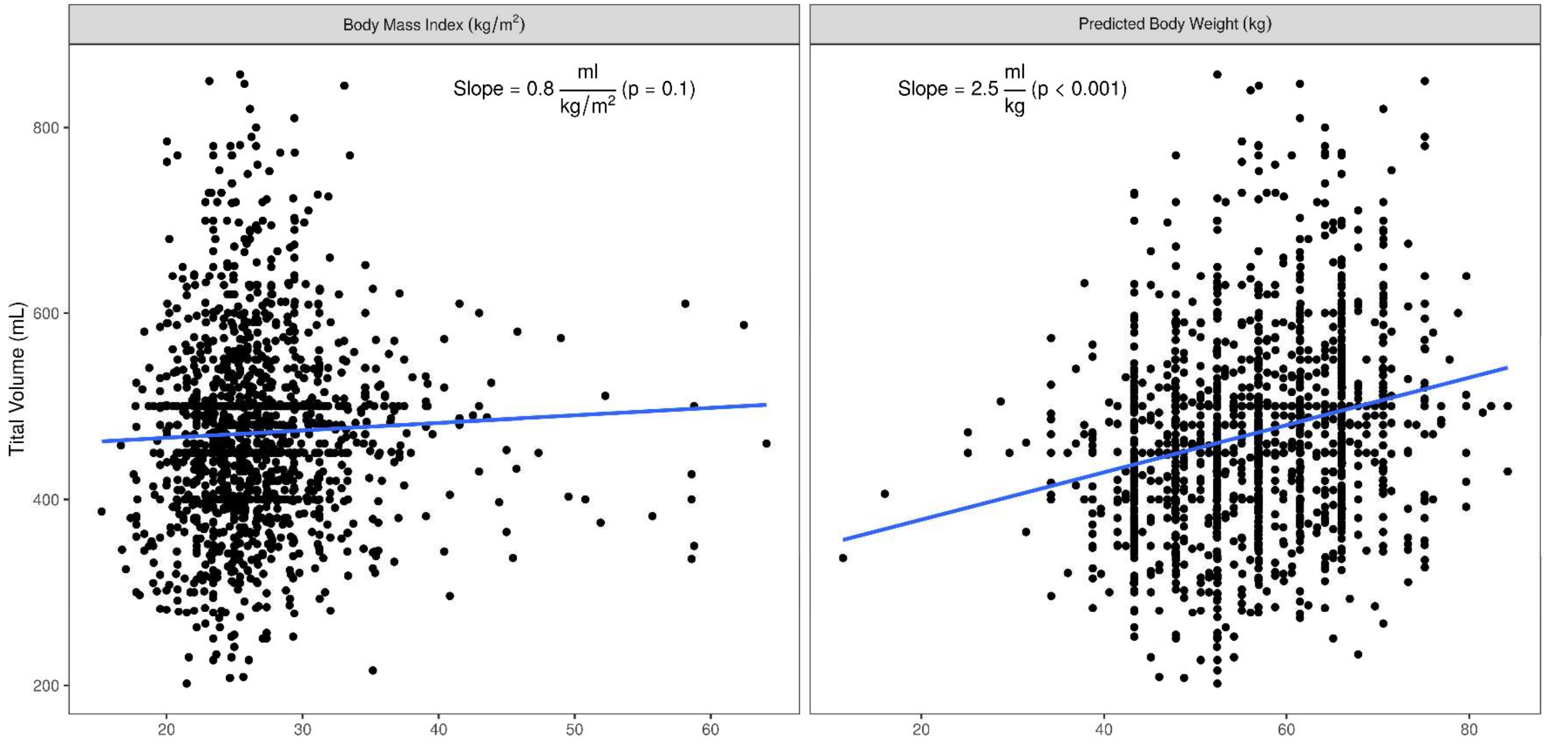

Figure S3
